# Cholesterol levels as a predictive marker for ICU survival in patients with cardiogenic shock supported by VenoArterial ExtraCorporeal membrane oxygenation

**DOI:** 10.1177/02676591251334896

**Published:** 2025-04-14

**Authors:** Maarten de Haan, Etienne Bertjens, Herman G Kreeftenberg, Mohamed A Soliman-Hamad, Rick Bezemer, R Arthur Bouwman

**Affiliations:** 1 Department of Anesthesiology, 3168 Catharina Hospital, Eindhoven, The Netherlands; 2 3169 BioMedical Diagnostics Lab. of the Signal Processing Systems Group of the Electrical Engineering Department of the Eindhoven University of Technology, Eindhoven, The Netherlands; 3 Department of Medicine, 1190 Vrije Universiteit Amsterdam, Amsterdam, The Netherlands; 4 Department of Intensive Care Medicine, 3168 Catharina Hospital, Eindhoven, The Netherlands; 5 Department of Intensive Care Medicine, Anna Hospital, Geldrop, The Netherlands; 6 Department of Cardiothoracic Surgery, 3168 Catharina Hospital, Eindhoven, The Netherlands

**Keywords:** veno-arterial extracorporeal life support, cardiogenic shock, cholesterol, ICU survival, prognostic marker

## Abstract

**Background:**

Veno-Arterial Extracorporeal Life Support (VA ECMO) is a critical intervention for patients with cardiogenic shock, serving as bridge to recovery, transplantation, or long-term therapies. The complexity of VA ECMO and its associated risks underscore the need for reliable prognostic markers to guide patient management. This study aimed to evaluate whether cholesterol levels could serve as a specific marker for ICU survival in patients with cardiogenic shock treated with VA ECMO.

**Methods:**

A retrospective observational study was conducted at Catharina Hospital Eindhoven, The Netherlands, between January 2013 and November 2019. Data from 67 patients treated with VA ECMO were analyzed. Cholesterol levels were measured daily from day 1 to day 5 after VA ECMO initiation. Demographic data, comorbidities, and outcomes were extracted from the patient data management system. Statistical analysis was performed, with a focus on non-normality of data distribution and the predictive value of cholesterol levels on ICU survival.

**Results:**

The study identified a significant association between higher cholesterol levels on the first day of VA ECMO treatment and increased ICU survival. A cholesterol threshold of 2.0 mmol/L was found to be an independent predictor of survival, with patients above this threshold having a higher survival rate. Multivariate logistic regression analysis confirmed the significance of this cholesterol threshold in predicting ICU survival.

**Conclusion:**

Cholesterol levels measured on the first day after the initiation of VA ECMO are a significant indicator of ICU survival in patients with cardiogenic shock. A threshold of 2.0 mmol/L is particularly predictive, offering a potential prognostic tool for clinicians managing these critically ill patients.

## Introduction

Veno-Arterial Extracorporeal Life Support (VA ECMO) is a sophisticated and highly effective technique employed to support patient circulation during cardiogenic shock.^1,2^ By providing both circulatory and pulmonary support, VA ECMO serves as a bridge to functional myocardial recovery, transplantation, or long-term therapies.^3^ The global utilization of VA ECMO is significant, with over fifty-eight thousand cases registered in the ELSO database, reflecting an Intensive Care Unit (ICU) survival rate of 47%.^4^ The outcomes for these patients, however, are heavily influenced by comorbidities and organ dysfunctions, which drive short-term mortality.^5^ Consequently, a multidisciplinary approach is imperative in daily practice, combined with diligent patient selection and timely application of VA ECMO.^6,7^ Therefore, several high-quality predictive scores exist to predict patient outcomes prior to initiating VA ECMO.^8–10^ However, the risk profile of patients changes significantly due to cardiac surgery combined with extracorporeal circulation and the subsequent need for circulatory support via VA ECMO. This shift in risk profile renders preoperative and pre-VA ECMO predictive scores potentially non-specific and unreliable. In contrast, the PREDICT VA-ECMO score is a validated tool that can inform daily treatment and management decisions for these patients.^11^

In the search for prognostic parameters in critically ill patients, cholesterol is an assessed parameter. Cholesterol is a combination of crucial lipids which plays multiple roles in human physiology, impacting signaling, immunity, sex hormones, and vitamin D synthesis,^12^ and is influenced by the condition of the patient. For example, in sepsis it has been recognized that hypocholesterolerolemia is associated with an increased risk of deterioration.^13–15^ In addition, in the context of cardiac surgery, low preoperative cholesterol levels are linked to an increased risk of postoperative sepsis,^16^ establishing cholesterol as a prognostic factor for outcomes in cardiac surgery patients.^17^ Therefore, the aim of this study is to investigate whether cholesterol levels serve as a specific marker for ICU survival in patients with cardiogenic shock supported by VA ECMO.

## Methods

### Data collection

Given the observational nature of this study, the local medical ethics committee granted approval and waived the requirement for informed consent (nWMO-2020.167). Data were extracted from the patient data management system (HiX™ Chipsoft, Amsterdam, the Netherlands) at Catharina Hospital Eindhoven, The Netherlands. The dataset from January 2013 and November 2019 contained demographic information, comorbidities, and laboratory values from patients with cardiogenic shock who were treated with VA ECMO. All patients initiated circulatory support via VA ECMO following a surgical intervention. Hypertension was defined according to the ESC/ESH guidelines.^18^ Diabetes Mellitus (DM) was recorded when patients were receiving insulin or other oral anti-diabetic medications according to the method of van Straten et al (2010).^19^ A patient was considered as having Chronic Obstructive Pulmonary Disease (COPD) when the patients were using medication for chronic pulmonary diseases. Peripheral Vascular Disease (PVD) was defined based on the criteria used by our department.^20^ Cholesterol levels were measured daily from day 1 to day 5 after the initiation of VA ECMO. Total cholesterol was determined during the morning routine check in the ICU. Patients were categorized into two groups based on their outcomes for further analysis.

### VA ECMO system and cannulation

The ECLS system utilized is a closed system comprising a centrifugal pump with an integrated oxygenator (Cardiohelp, Getinge GmbH, Rastatt, Germany) and a heater-cooler unit. Cannulation was performed in the femoral artery (19–21 French gauge) and femoral vein (23–25 French gauge), supplemented with a backflow catheter (6 French gauge) to ensure distal perfusion of the cannulated leg (V_f_-A_f_a ECMO).^21^ The cardiac surgeon carried out the cannulation either surgically or percutaneously, with the choice of method depending on the surgeon’s preference and the patient’s physical characteristics.

### ICU management

ICU management of VA-ELCS patients is a dynamic, patient-specific process throughout the duration of admission. Standard ICU monitoring includes arterial blood pressure, central venous pressure, heart rate, and oxygen saturation, along with cerebral Near-Infrared Spectroscopy. The patient is on artificial ventilation. Sedation during VA ECMO is usually managed by a combination of sedatives like propofol and opioids. After the initiation of VA ECMO, patients often experience a systemic inflammatory response syndrome which in turn requires fluid administration. The volume status of the patient is monitored by central venous oxygen saturation, clinical presentation, peripheral circulation, and trans esophageal echo (TEE). The unloading of the left ventricle is also checked via TEE, and if needed the unloading of the left ventricle is supported by Impella or IABP. Due to the placement of the supplemental back-flow catheter, the peripheral circulation of the canulated limp is checked every 2 hours. Thromboembolic prophylaxis is achieved through systemic anticoagulation with heparin, supplemented by Low Molecular Weight Heparin after drain leakage remains stable. For additional general patient treatment, the standard ICU care applies. This comprises for example head tilt 30° and ulcer prophylaxis.

### Statistical analysis

Initially, datasets were examined for skewness and kurtosis to evaluate the (non-)normality of the data distribution for each recorded variable. For normally-distributed continuous variables, data were presented as mean ± SD and groups were compared using the Student’s*t* test. Data which was non-normal distributed, were presented as median with interquartile range (IQR) (25%-75%), and comparisons between groups were made using the Mann-Whitney-U test. For categorical variables, data were presented as numbers and percentages, and comparisons between groups were made using the Fisher’s exact test. Univariate regression analysis was conducted to assess the potentially confounding effects of relevant biomedical and demographic factors on outcomes. Subsequently, significant confounders were included in the multivariate logistic regression analysis and due to the importance of sex, the multivariate regression is combined with sex. Odds Ratios (OR) with 95% Confidence Intervals (CI) were reported. For all statistical tests, a *p*-value of less than 0.10 was considered statistically significant. All statistical analyses were performed using SPSS version 25 (Chicago, IL, USA).

## Results

In this retrospective data study, we investigated patients with cardiogenic shock which were treated with VA ECMO and analyzed the cholesterol data gathered from Day 1 – 5 after the start of circulatory support.

### Patient population

Between January 2013 and November 2019, 81 patients with cardiogenic shock were treated with VA ECMO in the Intensive Care Unit. Of these 81 patients 14 patients were excluded from the analysis due to a low number of cholesterol measurements (*n* = 9) or an ICU stay of less than 48 hours (*n* = 5). Consequently, 67 patients were included in this retrospective observational study. The population was stratified based on the outcomes after 30-days. Demographic details of this cohort are presented in Table 1, while Table 2 provides an overview of the underlying conditions that necessitated VA ECMO therapy. The ICU stay for patients who survived the VA ECMO period was significantly longer (*p* < .001) compared to non-survivors, although a non-significant difference (*p* = .362) was observed in the duration of VA ECMO between the two groups. Comparisons of other variables did not reveal any additional statistically significant differences between the groups.

**Table 1.** Demographic and VA ECMO data.

Variables	Non-survivors (*n* = 38)	Survivors (*n* = 29)	*p*-value
Age	59.5 ± 11.8	58.8 ± 10.1	0.801
Gender (male)	25 (66%)	23 (79%)	0.280
BMI	26.0 (24.1–31.4)	27.7 (24.1–30.4)	0.422
Hypertension	17 (45%)	10 (34%)	0.457
Diabetes	5 (13%)	2 (7%)	0.689
COPD	4 (11%)	2 (7%)	0.691
PVD	2 (5%)	3 (10%)	0.655
Prior cardiac surgery	15 (39%)	6 (21%)	0.118
LVEF <35%	7 (18%)	7 (24%)	0.763
Preop creatinine	107.5 (82.0–142.0)	101.0 (84.0–159.5)	0.949
Logistic EuroSCORE	0.29 (0.04–0.66)	0.14 (0.07–0.49)	0.461
ICU stay (days)	7.0 (2.8–13.0)	16.0 (8.0–22.5)	<0.001
ECLS duration (days)	6.0 (1.8–12.0)	5.0 (2.0–7.5)	0.362

Data presented as mean ± SD, median (IQR) or number (%). BMI = body mass index in [kg/m^2^], COPD = chronic obstructive pulmonary disease, LVEF = left ventricle ejection fraction, preop creatinine in [µmol/L], and PVD = peripheral vascular disease.

**Table 2.** An overview of the conditions underlying the decision for VA ECMO.

Variables	Non-survivors (*n* = 38)	Survivors (*n* = 29)	*p*-value
Coronary surgery	1 (2.6%)	4 (13.8%)	0.158
Valve surgery	9 (23.7%)	11 (37.9%)	0.282
Combined surgery	8 (21.5%)	2 (6.9%)	0.168
Other	20 (52.6%)	12 (41.4%)	0.461

Data presented as number (%). In the Non-Survivors group patients were included in the valve surgery group after aortic valve replacement (AVR) (*n* = 3), mitral valve surgery (MVS) (*n* = 3) or combined AVR and MVS (*n* = 3). In the Survivor group this was AVR (*n* = 5), MVS (*n* = 4) or combined AVR and MVS (*n* = 1). Combined surgery consists of coronary surgery combined with AVR (*n* = 4), MVS (*n* = 2), and AVR + MVS (*n* = 2) in the Non-Survivor group. In the Survivors group this was coronary surgery combined with AVR (*n* = 1), and MVS (*n* = 3). Other cardiac causes in the Non-Survivor group were treated with VA ECMO due to Cardiac Arrest (*n* = 4), Ventricle Septum Rupture (*n* = 5), after aortic surgery (*n* = 5), Percutaneous Coronary Intervention (*n* = 1), and cardiomyopathy (*n* = 1). In the Survivors group the other causes for treatment with VA ECMO were Ventricle Septum Rupture (*n* = 4), Takotsubo (*n* = 1), Cardiac Arrest (*n* = 4), Influenza (*n* = 1), Cardiomyopathy (*n* = 1), Percutaneous Coronary Intervention (*n* = 1)

### Cholesterol levels in survivors and non-survivors over time

**Figure 1.**
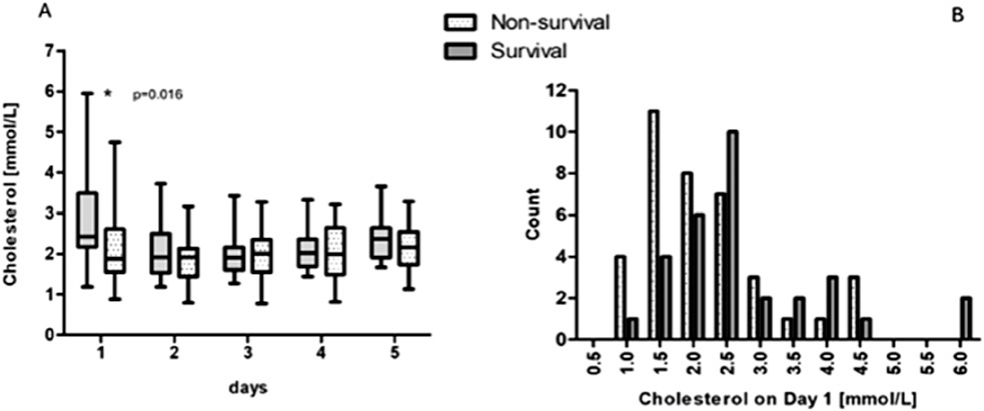
An overview of cholesterol determinations during five consecutive days (A), and on Day 1 (B).

Figure 1(A) illustrates the total cholesterol levels measured over five consecutive days following the initiation of VA ECMO treatment. On Day 1, the variability in cholesterol levels was the most pronounced, which then decreased and stabilized by Day 2, where the median cholesterol level reached the lowest point. After the initial drop from Day 1 to Day 2, cholesterol levels gradually increased over the subsequent days. On Day 1, cholesterol levels were significantly lower in the non-surviving group compared to the surviving group (1.87 [1.55–2.61] mmol/L vs 2.42 [2.17–3.49] mmol/L; *p* = .016).

### Cholesterol levels and their relation to outcome

**Figure 2.**
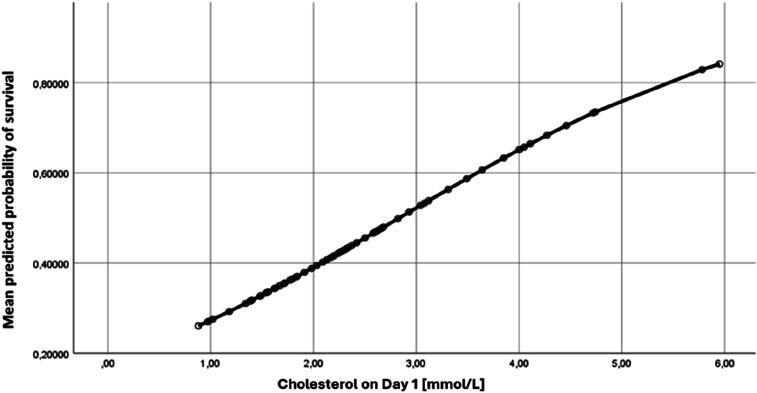
Predicted probability of survival based on the cholesterol level measured on Day 1 after starting VA ECMO treatment.

Figure 2 illustrates the predicted probability of ICU survival based on cholesterol levels measured on Day 1. Higher cholesterol levels on Day 1 are linked to a greater likelihood of survival. However, a cholesterol level ≤1.0 mmol/L on Day 1 showed that none of the patients survived the ICU. The ROC curve (Figure 2) for cholesterol on Day 1 after the initiation of VA ECMO shows an AUC of 0.669, highlighting the complexity of predicting outcomes in patients treated with VA ECMO.

**Figure 3.**
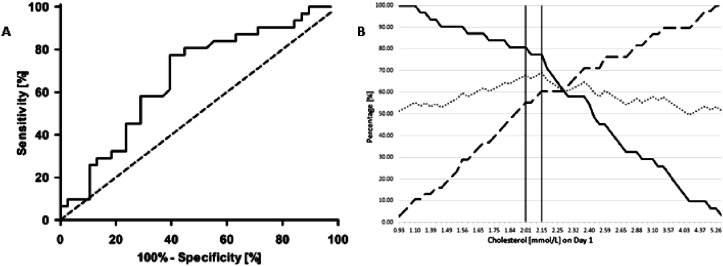
An overview of threshold determination in patients treated with VA ECMO. (A): Receiver Operating Curve of the minimal cholesterol level on Day 1 after starting with VA ECMO; (B) presents the cholesterol level [mmol/L] on Day 1, where the continuous line represents the sensitivity, the long-dashed line represents the specificity, and the dotted line represents the average of sensitivity and specificity. The verticals lines represents the discussed threshold of 2.0 and 2.15 mmol/L.

As shown in Figure 3(B), (a) cholesterol threshold on Day 1 of 2.15 mmol/L may be considered statistically optimal having the highest average, with a sensitivity of 77.42% and specificity of 60.53%. However, based on the histograms (Figure 1(B)), the predicted probability of survival curve (Figure 2), and the data underlying the ROC curve (Figure 3), we selected a clinically relevant cholesterol threshold on Day 1 of 2.0 mmol/L for further analysis. This threshold corresponds to a predicted ICU survival probability of 0.40, with a sensitivity of 80.7% and a specificity of 55.3% in the ROC curve. The survival rate for patients with cholesterol levels below 2.0 mmol/L (*n* = 25) was 20% (*n* = 5), whereas for those with levels at or above 2.0 mmol/L (*n* = 42), the survival rate was 59.5% (*n* = 25), (*p* = .002). Further analysis using univariate logistic regression indicated that a cholesterol level below 2.0 mmol/L on Day 1 was significantly associated with reduced ICU survival (OR 5.882; 95% CI 1.849-18.719; *p* = .003). All significant risk factors identified in the univariate analysis were included in the multivariate logistic regression model and combined with the variable sex, the results of which are presented in Table 3. This analysis confirmed that a cholesterol threshold of less than 2.0 mmol/L on Day 1 after the start of VA ECMO is a significant predictor of reduced ICU survival (OR 6.163; 95% CI 1.824-20.820; *p* = .003). Applying the cholesterol threshold of 2.0 mmol/L resulted in the highest OR related to survival of these patients.

**Table 3.** Univariate and multivariate analysis of variables for survival.

	Univariate analysis		Multivariate analysis	
Variables	Or (95% CI)	*p*-value	Or (95% CI)	*p*-value
Gender [male]	2.435 (0.799–7.420)	0.118	3.121 (0.932–10.455)	0.065
Age	1.027 (0.983–1.073)	0.227		
Diabetes	0.476 (0.112–2.028)	0.316		
Hypertension	0.893 (0.315–2.528)	0.831		
COPD	0.711 (0.155–3.252)	0.660		
PVD	1.318 (0.428–4.064)	0.631		
Preop creatinine	0.991 (0.980–1.002)	0.116		
LVEF <35%	0.321 (0.091–1.127)	0.076	0.225 (0.057–0.889)	0.033
Cholesterol^a^	1.725 (1.050–2.832)	0.031	1.894 (1.085–3.305)	0.025
Cholesterol <2.0^b^	5.882 (1.849–18.719)	0.003	6.163 (1.824–20.820)	0.003

Data presented as OR = odds ratio and 95% CI = confidence interval. Cholesterol in [mmol/L], COPD = chronic pulmonary obstructive disease, LVEF = left ventricle ejection fraction, preop creatinine in [µmol/L], and PVD = peripheral vascular disease.

^a^Entered as continuous variable on Day 1.

^b^Entered as dichotomous variable on Day 1.

## Discussion

VA ECMO is a highly effective technique for supporting the circulation in patients with severe cardiogenic shock. However, the complexity and associated risks make it burdensome for both patients and healthcare professionals. Therefore, identifying daily parameters that are not influenced by the ECLS system is crucial. This study aimed to determine if total cholesterol could serve as a predictive marker for ICU survival in patients with cardiogenic shock supported by VA ECMO.

Our findings indicate that cholesterol levels on the second and third days after initiating VA ECMO reached their lowest values. However, cholesterol levels measured on the first day were significantly different between survivors and non-survivors. Higher cholesterol levels on Day 1 were associated with increased survival chances. Both cholesterol as a continuous variable and a cut-off point of 2.0 mmol/L were found to be independent factors correlated with ICU survival. Thus, cholesterol appears to be a valuable parameter for assessing short-term outcomes in patients treated with VA ECMO for cardiogenic shock. Prognostication in these patients remains complex, hence we see a relatively low specificity for cholesterol solely as a prediction factor (55.3%). Events like liver failure, septic- or hemorrhagic shock, or leg ischemia could potentially influence markers.^28^ Combining multiple markers could lead to an improved specificity and therefore increased diagnostic accuracy in this complex population. An important marker of added value could be serum lactate level. A suggestion for future research is to combine biomarkers in statistical modeling strategies using Support Vector Machines for more accurate outcome prediction.^22^ This underscores the complexity of prognostication in patients treated with VA ECMO.

Total cholesterol includes Low-Density Lipoprotein (LDL), High-Density Lipoprotein (HDL), triglycerides, Very Low-Density Lipoprotein, and Lipoprotein A. It is relatively easy to determine and assess these parameters on a daily basis. Additionally, the cost of determining cholesterol is significantly lower than that of C-reactive protein and procalcitonin. Therefore, this study utilized total cholesterol as a prognostic indicator for ICU survival among patients with cardiogenic shock treated with VA ECMO. A decline in total cholesterol is often observed in critically ill patients, but it remains unclear whether this decline is due to deteriorating health or secondary to the illness.^14,17^ Several mechanisms have been proposed. In patients with low cholesterol levels, an increased mortality rate was observed. More specific, patients with 1.0 mmol/L showed a higher mortality rate compared to patients with a cholesterol level of 2.0 mmol/L. The reduction in total cholesterol can be attributed to: (1) increased intracellular metabolism in critically ill patients leads to the shutdown of less essential organ functions, inducing an anabolic state^23^; (2) reduced LDL efflux from the liver and increased uptake of LDL into cells^24,25^; (3) during critical illness, HDL particles assimilate serum amyloid A, an acute phase protein, causing a rapid increase in concentration during systemic inflammatory response syndrome (SIRS) reactions^26^; and (4) conflicting findings exist regarding whether triglyceride levels are elevated or reduced during critical illness.^25,27^ More severe illness can lead to a lower total cholesterol level and may be predictive of a higher mortality rate. On the contrary, high total cholesterol levels are also associated with a higher mortality rate. Chen et al (2023) suggested a U-shaped relationship between LDL-C levels and mortality risk, meaning that above and below a certain threshold mortality risk increases.^28^ Razavi et al (2023) described this similar U-shaped relationship between HDL-C levels and mortality risk.^29^ Regarding these relationships between LDL-C, HDL-C and an increased mortality rate, it is likely that the total cholesterol level shows a similar relationship explaining the higher mortality rate at 1.0 mmol/L compared to 2.0 mmol/L.

Regarding ICU survival, few studies have used this parameter as predictive marker. One of the studies by Vyroubal et al has shown that low cholesterol levels are correlated with sepsis.^30^ Moreover, a case series of five patients compared total cholesterol with procalcitonin and C-reactive protein as indicators of treatment success, finding that cholesterol could be a parameter for disease progression or recovery.^31^ These results support our findings in this study population.

Other research has confirmed the association between critically ill patients and cholesterol. Cholesterol has been assessed as a predictive factor for sepsis following general (trauma) surgery.^32^ Recent findings suggest that low cholesterol levels before elective cardiac surgery can differentiate between low- and high-risk patients. Additionally, cholesterol levels, both as absolute values and adjusted for hemodilution, remain stable during extracorporeal circulation (ECC) and at 3 hours and 24 hours post-surgery.^16^ Prolonged low cholesterol levels have been linked to organ failure, sepsis, and increased mortality rates.^16,17,30^ The findings of this study, aligning with previous research, suggest that low cholesterol levels are associated with adverse outcomes.

### Strengths and limitations

This report represents the initial analysis since the inception of our ECLS program in 2012. Given the retrospective nature of this study, certain limitations are inherent. Unknown confounding factors may have influenced the results. However, the data were generated in a highly efficient, well-organized cardiothoracic center, and protocol-driven center, enhancing the reliability of data interpretation. Preoperative cholesterol levels were not measured; thus, this study does not provide a complete overview of cholesterol levels throughout the VA ECMO period. In our ICU, cholesterol is used as a daily marker alongside standard markers, potentially offering physicians a more comprehensive understanding of the patient’s status.

The specificity in the ROC curve of the presented cholesterol threshold is low with 55.3%. An influencing factor of this low specificity could be the relatively small sample size in our study. Due to the complexity of prognostication in patients treated with VA ECMO, it would be of added value to perform future research with a large cohort of patients. Where the pre-VA ECMO prognostic scores could be influenced by the changing risk profile of the patients, for example due to elongated aortic cross-clamp times, other prognostic scores could potentially be impacted by the extracorporeal membrane oxygenator. Despite the relatively small sample size, this study contributes to the identification of predictive markers for patients undergoing VA ECMO.

The effects of small and heterogeneous groups were adjusted using multivariate regression analysis. Despite this, cholesterol remains a variable associated with ICU survival. The significantly longer ICU stay observed in survivors could be attributed to the extended recovery period post-VA ECMO, which includes challenges such as weaning off mechanical ventilation and regaining strength before transferring to the ward. Sedation of the patient is one of the pillars during the treatment with VA ECMO. The state of decreased stress and oxygen demand could be achieved with propofol. However, the vasodilating side-effect of propofol on top of the SIRS response could lead to additional administration of fluids or vaso-active medication (e.g., noradrenaline). The patients in our study were sedated with propofol, which contains 0.1 g/ml of lipids, and could potentially influence total cholesterol levels, leading to false-positive measurements. The potentially influencing factor of propofol on the total cholesterol levels was not within the scope of this study. Also, the use of cholesterol inhibitors (statins) preoperatively or during the VA ECMO period was outside the scope of this study. Furthermore, despite the relatively small sample size, this study contributes to the identification of predictive markers for patients undergoing VA ECMO.

## Conclusion

Our findings from this retrospective data study indicate that the total cholesterol level measured on the first day after the initiation of VA ECMO is an indicator associated with ICU survival in patients undergoing VA ECMO due to cardiogenic shock. Higher cholesterol levels are linked to better survival outcomes. Specifically, a cholesterol threshold of 2.0 mmol/L has been identified as an independent predictor of ICU survival.

## Data Availability

Data is available upon request from the corresponding author.*
